# Case Report: Charcot-marie-tooth disease caused by a *de novo MORC2* gene mutation - novel insights into pathogenicity and treatment

**DOI:** 10.3389/fgene.2024.1400906

**Published:** 2024-10-11

**Authors:** Feng Zhu, Chengcheng Gao, Xiangxiang Zhu, Huihua Jiang, Mingchun Huang, Yuanlin Zhou

**Affiliations:** ^1^ Department of Neurology, Taizhou Hospital of Zhejiang Province Affiliated to Wenzhou Medical University, Taizhou, China; ^2^ Zhejiang Key Laboratory of Digital Technology in Medical Diagnostics, Dian Diagnostics Group Co., Ltd., Hangzhou, China; ^3^ Supply-Room, Taizhou Hospital of Zhejiang Province Affiliated to Wenzhou Medical University, Taizhou, China

**Keywords:** *MORC2*, lower limb weakness, charcot-marie-tooth disease, mecobalamin, coenzyme Q10, case report

## Abstract

Charcot-Marie-Tooth disease (CMT) is a hereditary peripheral neuropathy involving approximately 80 pathogenic genes. Whole-exome sequencing (WES) and confirmatory Sanger sequencing analysis was applied to identify the disease-causing mutations in a Chinese patient with lower limb weakness. We present an 18-year-old male with a 2.5-year history of progressive lower limb weakness and an unsteady gait. Upon admission, a physical examination revealed hands tremulousness, bilateral calf muscle wasting and weakness, pes cavus, and elevated serum creatine kinase (CK) levels. Electromyography demonstrated axonal neuropathy affecting both upper and lower limbs. A *de novo* heterozygous missense mutation was identified in the *MORC2* gene, NM_001303256.3: c.1199A>G, NP_001290186.1: p.Gln400Arg. Consequently, these clinical and genetic findings suggested a diagnosis of hereditary peripheral neuropathy, CMT type 2Z. Oral mecobalamin and coenzyme Q10 was initiated as subsequent treatment. Our study firstly reports the *MORC2* c.1199A>G mutation occurring *de novo*, highlighting its causal association with CMT2Z, and prompting its reclassification as likely pathogenic. Oral mecobalamin and coenzyme Q10 might be a potential treatment approach for early-stage CMT2Z. We recommend genetic testing for CMT patients to identify the genetic etiology, thereby improving clinical management and facilitating genetic counseling.

## 1 Introduction

Charcot-Marie-Tooth disease (CMT) is the most prevalent inherited peripheral neuropathy, with an incidence of 1 in 2,500 individuals (Fridman and Saporta, 2021). It is characterized by chronic motor and sensory polyneuropathy that manifests progressive, symmetrical distal muscle weakness and atrophy, diminished ankle dorsiflexion strength, depressed tendon reflexes, and pes cavus. CMT has significant genetic and clinical heterogeneity (Juneja et al., 2019; Volodarsky et al., 2021). Presently, over 80 genes have been implicated in various CMT subtypes (Stavrou and Kleopa, 2023). Although CMT can occur at any age, it predominantly arises between the first and third decades, severely impairing patients’ social participation and life quality from an early stage (Stavrou and Kleopa, 2023; Hertzog and Jacob, 2023). Therefore, elucidating the genetic etiology of CMT is critical for precision diagnosis, classification of subtypes, timely intervention, and genetic counseling for subsequent generations.

*MORC2*, encoding the microcheilia CW-type zinc finger protein 2, has been identified as the causative gene for CMT type 2Z (CMT2Z, MIM: #616688). CMT2Z is an autosomal dominant axonal peripheral neuropathy that initially presents with weakness and sensory impairment in the distal lower limb muscle and progressively involves the upper limbs and proximal muscle, leading to severe disability (Sevilla et al., 2016; Ando et al., 2017). However, the association between specific *MORC2* mutations and the manifestation of CMT2Z remains controversial due to the limited patient population and the absence of functional assay. Further research is required to clarify this association.

In this study, we investigated a patient presenting with peripheral neuropathy. Utilizing whole-exome sequencing (WES) followed by Sanger sequencing, we identified a *de novo MORC2* c.1199A>G mutation in the proband, leading to an upgrade in its mutation classification. Additionally, we detailed the proband’s disease progression and treatment approach, providing a comprehensive view on the clinical management of CMT2Z.

## 2 Case description

### 2.1 Case presentation

**FIGURE 1:**
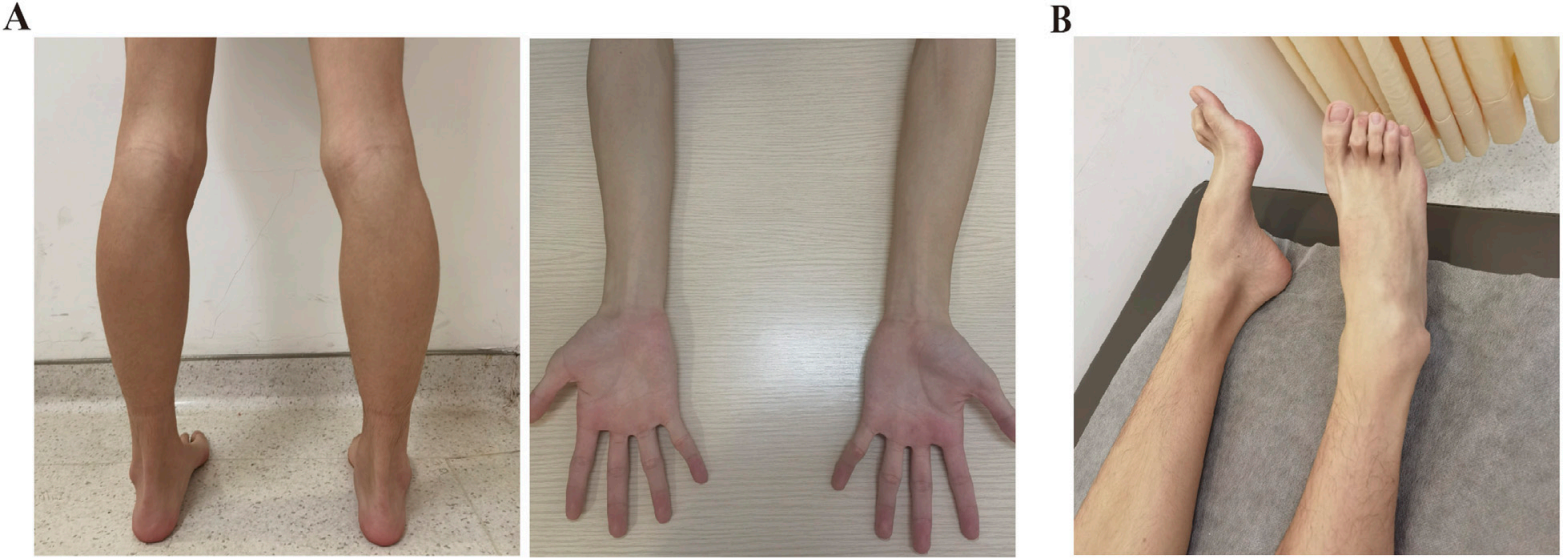
Clinical manifestations of the patient. **(A)** Physical examination revealed bilateral calf muscle wasting and slender arms. **(B)** Pes cavus was observed in the patient.

An 18-year-old Chinese Han male was admitted to Taizhou Hospital for a 2.5-year history of lower limb weakness and an unsteady gait. The patient presented with a generally lean body. Physical examination revealed decreased tendon reflex with a negative Babinski sign and intact cranial nerve function. Tremulousness was observed when extending his hand. Wasting of the bilateral calf muscle was observed, with muscle strength in the distal lower limbs rated at 4/5 (see Table 1; Figure 1A). Strength in the neck muscles, both proximal and distal upper limbs muscle, and proximal lower limbs muscle was normal and rated at 5/5. He was unable to perform heel walking due to bilateral dorsiflexion weakness of the feet, and a significant high arch was observed (Figure 1B). The patient didn’t report any hearing loss, decreased vision, or significant limb paresthesia. Notably, he experienced low physical achievement and a slower running speed since childhood (Supplementary Figure S1). His physical and intellectual development were normal, with a height of 1.68 m, a weight of 45 kg, and moderate academic performance in university. All immediate family members, including his parents (Ⅰ-1 and Ⅰ-2, aged 42) and younger sister (Ⅱ-2, aged 7), did not report any limb weakness. The physical assessments of the three family members revealed normal motor skills, characterized by an unimpaired gait and running posture, as well as normal arches and tendon reflexes, indicating their overall health. Given their unimpaired motor competencies, the family members declined further examinations.

**TABLE 1:** Summarized clinical features of patients carrying MORC2 c.1199A>G.

Clinical features	This study	Ando et al. (2017)	Zhao et al. (2016)		
		No. 9	Ⅲ-5	Ⅲ-6	Ⅱ-5
Gender	Male	Male	Male	Male	Female
Age at exami/tion	18	44	34	30	62
Age at onset	16	6	15	12	30
Initial symptoms	Lower extremity weakness and an unsteady gait	Poor motor performance	Distal lower limbs weakness	Distal lower limbs weakness	Distal lower limbs weakness
Upper limbs involvement	Yes	Yes	Yes	Yes	Yes
Proximal involvement	No	No	Yes	Yes	Yes
Laterality	Bilateral	Bilateral	Bilateral	Bilateral	Bilateral
Muscle weakness	Distal lower limbs	Distal leg weakness	Distal and proximal limbs (upper and lower), pelvic and shoulder girdle	Distal and proximal limbs (upper and lower), pelvic and shoulder girdle	Distal and proximal limbs (upper and lower), pelvic and shoulder girdle
Muscle atrophy	bilateral calf muscle	Lower limbs	Distal and proximal limbs (upper and lower), pelvic and shoulder girdle	Distal and proximal limbs (upper and lower), pelvic and shoulder girdle	Upper and lower limbs
Sensory disturbance	No	Decrease	Vibratory	Vibratory	—
Vibration	—	Decrease	Decrease	Decrease	—
Hand tremor	Yes	—	Yes	Yes	—
Pes cavus	Yes	—	Yes	Yes	—
Gait	Unsteady	—	Steppage	Steppage	—
Tendon reflex	Decrease	Decrease	Absent	Absent	—
Babinski sign	Negative	—	—	—	—
Cranial nerve examination	Negative	—	—	—	—
Cognitive impairment	No	—	—	—	—
Hearing loss	No	—	—	—	—
Creatine kinase (initial evaluation)	539 U/L (50∼310)	—	—	—	—
Electrophysiology	MNCV and upper limbs SNAP reduced, distal lower limbs SNAP and SNCV absent, lower limbs mean F-wave latency extended	Axonal type	CMAP amplitude reduced, SNAP and SNCV absent	CMAP amplitude and MNCV reduced, SNAP and SNCV absent	—
family history	Sporadic	AD	AD		

MNCV, moto nerve conduction velocity; SNAP, sensory nerve action potential; SNCV, sensory nerve conduction velocity; AD, autosomal dominant inheritance.

The patient showed an elevated serum CK at 539 U/L, which is above the normal range of 50 to 310 U/L. Electromyopraphy (EMG) study performed using Keypoint 9033A07 showed peripheral nerve axonopathy in both the upper and lower limbs, evidenced by a decreased motor nerve conduction rate and reduced amplitude of sensory nerve potentials in the upper limbs (see Table 2). Additionally, there was an absence of potential amplitude in the bilateral superficial peroneal and sural nerve, along with an extended mean F-wave latency in the bilateral tibial nerve (see Table 2). All these findings collectively indicate peripheral neuropathy in the patient.

**TABLE 2:** Electrophysiological data of the proband.

	Latency, ms				Amplitude, mV		Conduction velocity, m/s	
	Left	Right			Left	Right	Left	Right
Compound muscle action potential								
Ulnar nerve								
Wrist - ADM	2.71	2.26			7.8	7.7	—	—
Elbow - Wrist	8.00	7.38			6.0	7.2	47.8^a^	48.8^a^
Median nerve								
Wrist - APB	3.65	3.44			7.5	9.3	—	—
Elbow - Wrist	7.81	7.69			6.1	8.4	51.7	53.6
Tibial nerve								
Ankle - AH	3.54	3.65			5.2	7.0	—	—
Popliteal fossa - Ankle	14.30	14.50			4.5	5.2	37.2^a^	36.9^a^
Common peroneal nerve								
Ankle - EDB	4.71	4.46			3.0	4.5	—	—
Fibular Head - Ankle	13.60	13.60			2.5	3.7	36.0^a^	33.7^a^
Sensory nerve action potential								
Ulnar nerve								
Wrist - Finger Ⅴ	2.85	2.82			0.8*10^–3^ #	2.5*10^–3^ ^a^	53.9	54.8
Radial nerve								
Thenar eminence - Wrist	1.99	2.12			2.9*10^–3^ #	5.8*10^–3^ ^a^	62.1	53.7
Median nerve								
Finger Ⅰ - Wrist	2.48	2.50			4.1*10^–3^ #	5.6*10^–3^ ^a^	63.1	61.5
Finger Ⅲ - Wrist	3.00	3.02			4.8*10^–3^ ^a^	5.5*10^–3^ ^a^	61.1	60.1
Superficial peroneal nerve								
LLL - FD	loss^a^	loss^a^			—	—	—	—
Sural nerve								
posterior LLL - LM	loss^a^	loss^a^			—	—	—	—
F-wave detection	Minimum F latency, ms				F-M mean latency, ms		Mean F latency, ms	
Median nerve								
Wrist - APB	25.1	25.7			21.3	22.0	26.0	26.9
Tibial nerve								
Ankle - AH	58.8	56.9			55.1	53.8	59.8^a^	58.4^a^

ADM, abductor digiti minimi; APB, abductor pollicis brevis; AH, adductor hallucis; EDB, extensor digitorum brevis; FD, foot dorsum; LLL, lateral lower leg; LM, lateral malleolus.

^a^ Abnormal value.

**FIGURE 2:**
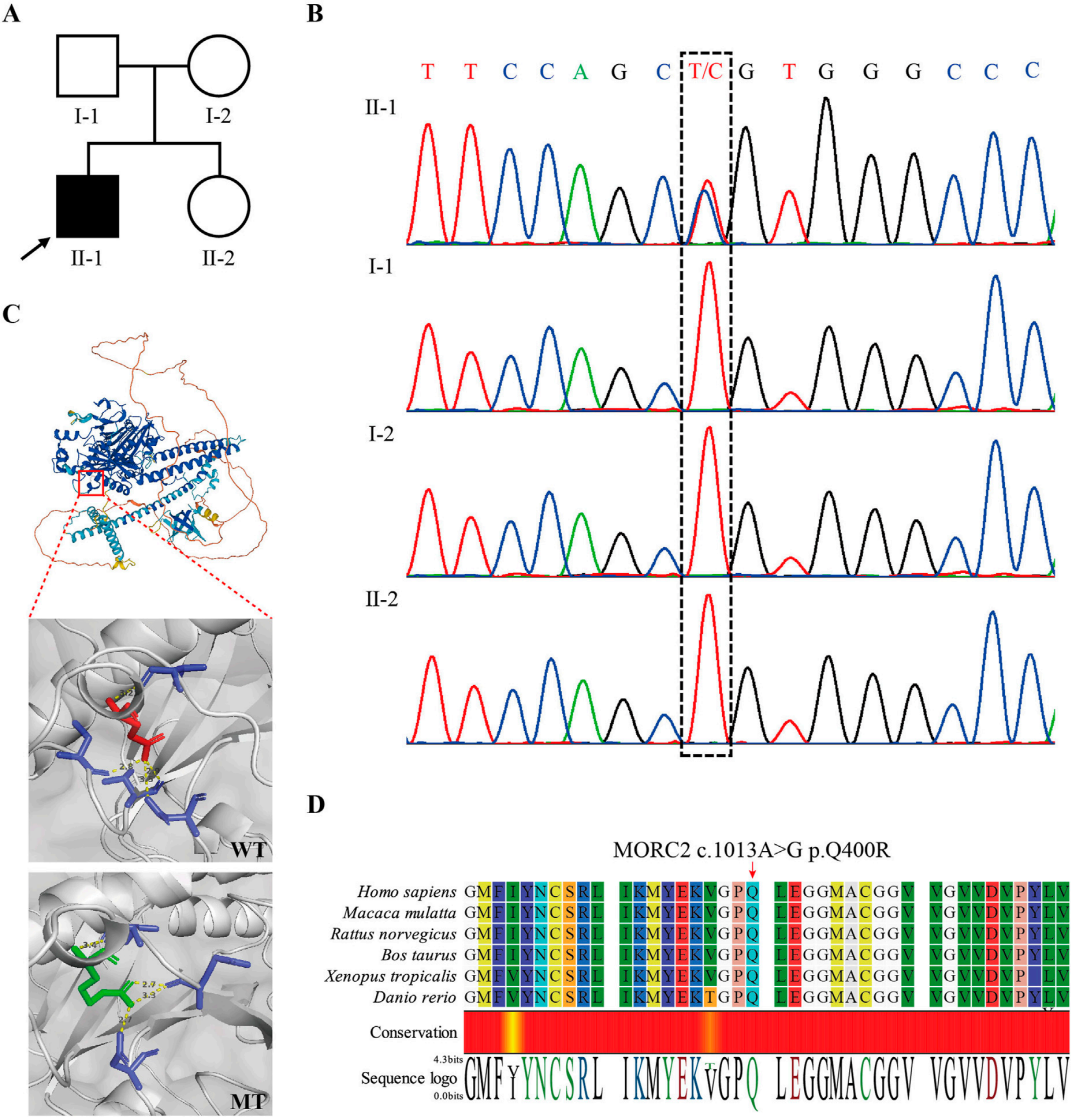
Identification of *MORC2* NM_001303256.3: (C)1199A>G, p.Q400R in the patient. **(A)** Pedigree of the presented family. The arrow indicates the proband. **(B)** Sanger sequencing confirmed the *de novo MORC2* c.1199A>G mutation in the proband. **(C)** 3D structure modeling shows the location of *MORC2* p.Q400R. The *MORC2* p.Q400R mutation alters the location and length of hydrogen bonds at the 400 residue. The wildtype Morc2 is predicted to form hydrogen bonds with the 380 Asp, 397 Val, 407 Cys, and 410 Val residues at the 400 Glu residue, while the mutant Morc2 is predicted to form hydrogen bonds with the 380 Asp, 395 Glu, and 397 Val residues at the 400 Arg residue. The Red amino acid indicates the Glu and green amino acid indicates the Arg. WT, wide type. MT, mutant type. **(D)** Evolutionary conservation of amino acid position p.Q400R in Morc2 protein.

To determine the genetic cause of peripheral neuropathy in this patient, we conducted WES on peripheral blood samples collected from the patient (II2). We identified a heterozygous missense mutation, *MORC2* NM_001303256.3: c.1199A>G, p.Gln400Arg, located within the 13th exon of the *MORC2* gene. Subsequent Sanger sequencing of the parents revealed that *MORC2* c.1199A>G was a *de novo* mutation (meet ACMG evidence PS2) (Figures 2A, B). This mutation was not recorded in the Genome Aggregation Database (GnomAD, http://gnomad-sg.org/) (meet ACMG evidence PM2_supporting), while it has been previously identified in two independent probands diagnosed with autosomal dominant CMT2, being classified as of uncertain significance in the ClinVar database (also recorded as NM_014941.1: c.1013A>G, p.Q388R) (meet ACMG evidence PS4_Moderate) (Ando et al., 2017; Zhao et al., 2016). Predictions from computational programs such as REVEL, ClinPred, and MAGPIE were applied (Liu et al., 2024). The REVEL score was 0.411 (benign supporting), while the ClinPred and MAGPIE scores were 0.991 and 0.807, respectively (both indicating pathogenicity support). SIFT and Polyphen2 indicated that *MORC2* p.Q400R is damaging and possibly damaging, respectively. An evolutionary conservation analysis showed that the *MORC2* p.Q400 site is highly conserved (Figure 2D). 3D structure modeling suggested that *MORC2* p.Q400R causes a change in the amino acid residue involved in the hydrogen bonds with 400 Glu residue, potentially impacting the Morc2 protein structure and stability (Figure 2C). Considering these results and following the ACMG guidelines (Richards et al., 2015), *MORC2* c.1199A>G mutation’s classification can be upgraded to likely pathogenetic (PS2+PS4_Moderate + PM2_supporting). No additional rare pathogenic variants were detected in any other genes associated with neuromuscular disease.

Therefore, the combination of clinical manifestations and genetic analysis supported a diagnosis of CMT2Z in this patient, which is associated with the *MORC2* c.1199A>G mutation. The patient had been prescribed vitamin B1 at a dose of 10 mg three times daily for a short period but discontinued it upon hospital admission. Considering that CMT2Z is pathophysiologically caused by axonal lesions, oral methylcobalamin and coenzyme Q10 (CoQ10) were initiated for subsequent treatment, at doses of 0.5 mg three times daily and 10 mg three times daily, respectively. As of the latest update 7 months post-diagnosis, the patient’s CK levels have normalized to 227 U/L, and he has not reported any further physical symptoms. Monitoring of the patient’s CK level and motor abilities will continue to determine the long-term effectiveness of the treatment and to manage his condition.

Furthermore, the proband’s younger sister doesn’t carry the mutation, suggesting a low risk of her developing similar symptoms. Nonetheless, prenatal diagnosis is recommended for future pregnancies in this family to prevent the potential transmission of a mutated allele, especially considering the possibility of gonadal mosaicism in a parent. Additionally, the proband could benefit from third-generation *in vitro* fertilization before pregnancy to prevent the inheritance of this disease.

### 2.2 Genetic testing methodology

Genomic DNA was exacted from 5 mL of patient’s peripheral blood using the QIAamp DNA Blood Mini Kit (Qiagen, Germany) and was subsequently fragmented into 180–280 bp segments with a Covaris LE220R-plus (Covaris, USA). A sequencing library was constructed, including end repair, phosphorylation, A-tailing, and adapters ligation. Then the exome enrichment was performed using the xGen Exome Research Panel v1.0 Kit (Integrated DNA Technologies, USA). The sequencing library underwent PCR amplification followed by sequencing on the NovaSeq 6000 platform (Illumina, USA). The overall average sequencing depth is 511×, with 99.1% of variants were covered at a depth greater than 20×.

The raw sequence data were firstly filtered to remove adapter sequences and low-quality reads. The remaining high-quality clean data were aligned to the GRCh37/hg19 human reference genome using the Burrows-Wheeler Aligner (BWA) tool (Li and Durbin, 2010). The variants calling was performed with the Genome Analysis Toolkit (GATK) (McKenna et al., 2010), followed by annotated according to the 1000 Genomes Project, GnomAD, ClinVar, and dbNSFP databases. Candidate mutations, were filtered based on allele frequency (AF < 1%), phenotypic concordance with the MIM database, inheritance patterns, and pathogenicity classification (pathogenic and likely pathogenic) according to ACMG guidelines (Richards et al., 2015).

Candidate variants were validated using Sanger sequencing. The primer sequences for *MORC2* c.1199A>G were as follows: forward 5′-AGC​TCT​CCG​GCG​TTT​GTA​AC-3′; reverse 5′-ACA​TTG​AAC​ACC​GGG​ATC​TGG-3′. PCR amplicons were subsequently sequenced on an ABI 3130 Genetic Analyzer (Applied Biosystems, California).

## 3 Discussion

With the advancement of genome sequencing technology, an increasing number of CMT causal genes have been discovered. *MORC2* was initially identified in a clinical cohort with axonal lesions and was reported to be associated with CMT in 2016 (Sevilla et al., 2016). The *MORC2* gene is located on chromosome 22q12.2 in humans and encodes a DNA-dependent ATPase consisting of 26 exons and 1,032 amino acids (aa). The protein contains three functional domains: Histidine kinase/HSP90-like ATPase superfamily (23∼278aa), Morc S5 domain 2-like (330∼451aa), and CW-type zinc finger (490∼544aa), and is involved in epigenetic silencing, genome homeostasis, transcriptional regulation, and lipid metabolism (Guillen Sacoto et al., 2020; Sanchez-Solana et al., 2014; Li et al., 2012; Douse et al., 2018). To date, 15 *MORC2* mutations have been identified in CMT2Z patients (Jacquier et al., 2022). Some of the functional impacts of these mutations have been elucidated. For instance, the *MORC2* p.R252W mutation has been reported to hyper-activates Human Silencing Hub (HUSH)-mediated transcriptional repression and perturbs ATPase dimerization dynamics in neuronal cells (Douse et al., 2018; Tchasovnikarova et al., 2017). Another hotspot mutation, *MORC2* p.S87L, has been shown to impair the proliferation of induced pluripotent stem cells (iPSCs) by inhibiting the MAPK/ERK and PI3K/Akt pathways, along with profiles of differentially expressed genes (DEGs) between p.S87L and p.Q400R (Zeng et al., 2024). Based on the above published transcriptomics data (Zeng et al., 2024; Mu et al., 2024), we investigated the DEGs between healthy controls and p.Q400R (Supplementary Figure S2). Kyoto Encyclopedia of Genes and Genomes (KEGG) analysis revealed significant enrichment of the MAPK signaling pathway in moto neuron precursor cells (MNPs) (Supplementary Figure S3), a pathway also enriched in iPSCs harboring the p.S87L mutation. However, no significant enrichment of pathways associated with cell proliferation and differentiation was observed in iPSCs carrying the p.Q400R. This suggests that *MORC2* p.Q400R mutation may dysregulate the intracellular and extracellular signaling pathways, such as MAPK signaling cascade, adversely affecting motor neuron function post-differentiation and ultimately contributing to the development of CMT2Z. Despite these findings, further research is still required to clarify the molecular mechanisms by which *MORC2* mutations lead to neuronal cell dysfunction and the onset of CMT.

*MORC2* exhibits significant phenotypic heterogeneity and a wide mutation spectrum. *MORC2*-associated disorders range from CMT2Z to symptoms resembling spinal muscular atrophy (SMA), with early onset and predominantly proximal muscle involvement. More severe neurodevelopmental conditions, characterized by developmental delay, impaired growth, dysmorphic facies, and axonal neuropathy (known as DIGFAN, MIM: #619090), have also been reported (Guillen Sacoto et al., 2020; Duan et al., 2021a; Lassuthova et al., 2016). It is noteworthy that all reported *MORC2* mutations are missense, with a high missense Z score of 3.23 (Stafki et al., 2023; Sivera et al., 2021). One possible explanation for the predominance of missense mutations may be the high conservation and dosage sensitivity of the Morc2 protein, where more deleterious alterations such as nonsense or frameshift mutations, and exon deletions could result in embryonic lethality, thus making them unobservable in live births. According to GnomAD, *MORC2* has a loss-of-function intolerant pLI score of 1 and a low observed/expected (oe) ratio of 0.1 (90% CI 0.06∼0.2), suggesting a high deleterious of virulent mutations. To date, no *MORC2*-knockout animal models have been developed, except for a mouse model harboring a Morc2a Ser87-to-Leu (p.S87L) knockin mutation (Lee et al., 2021). Additionally, the distribution of the *MORC2* mutations does not exhibit a specific pattern correlating with symptom severities, mainly located on the ATPase, coiled-coil domain 1, and ribosomal protein S5 domain at the N-terminus (Jacquier et al., 2022; Duan et al., 2021a). However, mutations at different positions have varying effects on protein function. For instance, the *MORC2* p.S87L and p.R252W mutations decrease Morc2 ATPase activity and hyperactivated HUSH-mediated epigenetic silencing, whereas the *MORC2* p.T424R mutation has the opposite effect (Tchasovnikarova et al., 2017; Duan et al., 2021a). Furthermore, whether the mutation is inherited or *de novo* appears to influence phenotypes. A *de novo MORC2* mutation is frequently associated with more severe manifestations, often involving central nervous system (CNS) disorder, while it is less common in CMT2Z patients. This discrepancy may be attributed to physiological or social infertility that naturally blocks the propagation of severely symptomatic *MORC2* mutations to subsequent generations. Therefore, it is essential to pay close attention to disease progression for patients identified *de novo MORC2* mutation, to facilitate timely disease-modifying interventions. Collectively, *MORC2* has a broad mutation spectrum and phenotypic variability, rendering the disease subtype classification and clinical management of patients challenging.

In this study, we report for the first time a heterozygous missense, *MORC2* c.1199A>G, occurring *de novo* in a young male with peripheral nerve axonopathy. Our study prompts a reclassification of this mutation from VUS to Likely Pathogenic, supporting the goal set by the National Human Genome Research Institute (NHGRI) to resolve all VUS in coding regions of the human Genome by 2030 (Green et al., 2020). Additionally, this mutation, also recorded as NM_014941.3: c.1013A>G, p.Gln338Arg, has been previously identified in four CMT-afflicted patients, three from a Chinese family and one from a Japanese family (see Table 1) (Ando et al., 2017; Zhao et al., 2016). Our study corroborates that individuals harboring *MORC2* c.1199A>G mutations predominantly exhibit peripheral neuropathy symptoms with a low risk of CNS involvement. Furthermore, our study, together with previous reports of *de novo MORC2* mutations, highlights the high mutation rate of the *MORC2* gene. While the recurrence of the *MORC2* c.1199A>G mutation suggests a potential hotspot within the East Asian CMT2Z cohort, further investigation such as haplotype analysis, is required to confirm this hypothesis.

Compared with previously reported four patients carrying *MORC2* c.1199A>G mutation, we conducted a detailed study of the clinical features of this mutation (see Table 1) (Ando et al., 2017; Zhao et al., 2016). Patients typically develop bilateral lower limb weakness and muscle atrophy in childhood or adolescence, with subsequent progression to the proximal lower limbs and eventually the upper limbs. This progression is often accompanied by pes cavus, a pathologic gait, sensory disturbance, and areflexia. However, variability in clinical manifestations was also observed, particularly in gender differences. The female patient presented milder manifestations than her brothers, including a later onset at age 30 and fewer involved muscle groups. Similar gender-specific differences in symptom severity have also been observed in other neuromuscular diseases, such as *SCN4A* mutation-associated hypokalemic periodic paralysis, suggesting a potential area for further investigation of CMT2Z (Li et al., 2012). Notably, a previous study has reported elevated serum CK levels in *MORC2*-associated CMT(24). Similarly, our patient also showed a significant elevation in CK levels during the initial evaluation, approximately twice the upper limit of the normal range. Since muscle atrophy is a common progression in CMT2Z, we suggest longitudinal monitoring of CK levels might reflect muscle status to a certain extent, particularly during therapeutic and rehabilitative interventions. However, it is worth noting that using CK alone to assess disease progression and treatment efficacy is limited, as it can be affected by many factors, such as physical activities, alcohol abuse, and intramuscular injections.

Notably, EMG revealed an absent sensory nerve action potential (SNAP) amplitude of the superficial peroneal nerve and sural nerve, while the patient denied any sensory abnormalities. This discrepancy may be attributed to the high degree of clinically heterogeneity in CMT disease. In a cohort study involving 465 unrelated Chinese CMT patients, four patients exhibited absent sensory nerve action potential (SNAP) amplitude and conduction velocity of the sural nerve, ulnar nerve, and median nerve, while three of them still retained decreased sensory vibration and the other one retained decreased sensory pinprick (Duan et al., 2021b). In another study, a CMT patient denied any sensation symptoms despite neurophysiological examination showing slight abnormalities in EMG and impairment of sensory fibers in the peripheral nerves of all four limbs (Cao et al., 2023). Overall, electrophysiological results may significantly mismatch with clinical manifestations in CMT disease. Considering this findings, the patient’s tolerance to partial sensory loss may also contribute to the discrepancy between the EMG results and clinical presentation in our study. The difficulty in obtaining biopsy materials also prevents us from further verifying the specific damage to the sensory nerves.

Current therapeutic approaches for CMT are symptomatic and focus on rehabilitation such as strength training of proximal and core muscles, the application of foot orthoses, and anticonvulsants for managing positive sensory symptoms (Yiu et al., 2022; Corrado et al., 2016; Magy et al., 2018). Reports suggest that low-dose methylprednisolone tablets and energy supplements can help to reduce serum CK levels, while vincristine may induce neurotoxicity in patients with CMT2Z (Vujovic et al., 2021; Wang et al., 2022). In our study, Oral mecobalamin and CoQ10 were administered for subsequent treatment. Mecobalamin, an active form of vitamin B12, has been widely used as a supportive intervention in various peripheral neuropathies due to its capacity for neuronal preservation and facilitation of injured nerve regeneration (Xiong et al., 2022; Sawangjit et al., 2020). CoQ10, a small lipid-soluble benzoquinone, is a crucial source of indispensable endogenous antioxidants. CoQ10 has demonstrated protective properties against vincristine-induced peripheral neuropathy and assists in managing *COQ7* gene mutation-associated distal hereditary motor neuropathy (Elshamy et al., 2022; Jacquier et al., 2023). Both mecobalamin and CoQ10 have been substantiated for safety (Sawangjit et al., 2020; Hathcock and Shao, 2006). After 7 months, the serum CK level normalized from 539 U/L to 227 U/L, suggesting a potentially treatment approach for early-stage CMT2Z. However, whether the decreased serum CK is due to this treatment is less clear and needs long-term follow-up and multiple perspectives assessment. Novel therapies, including gene therapy and targeted pharmaceuticals such as NRG-1 axis inhibitors and modulators of unfold protein response (UPR) and histone deacetylase (HDAC) enzyme family, are currently in development and promise to provide more precise treatment for CMT patients in the near future (Stavrou and Kleopa, 2023; Hertzog and Jacob, 2023; Beloribi-Djefaflia and Attarian, 2023). We will continue to monitor the proband’s disease progression and remain updated about the latest treatment advancements to improve his quality of life.

In summary, our study describes a male adolescent patient with peripheral neuropathy carrying a *de novo* heterozygous missense mutation, *MORC2* c.1199A>G. Our findings further support the causal association between this mutation and CMT2Z, upgrading its mutation classification to likely pathogenic. Oral mecobalamin and CoQ10 appear to be a potential treatment option for early-stage CMT2Z patients but the efficacy of this therapy needs further evaluation. We emphasize the importance of genetic testing for individuals with CMT to determine the genetic etiology, which can significantly improve clinical management and provide valuable insights for genetic counseling.

## Data Availability

The original contributions presented in the study are included in the article/Supplementary Material, further inquiries can be directed to the corresponding author.
