# Supplementary material for: Case Report: Charcot-marie-tooth disease caused by a de novo MORC2 gene mutation - novel insights into pathogenicity and treatment
Source: Front Genet. 2024 Oct 11;15:1400906. doi: 10.3389/fgene.2024.1400906 (PMC11512448; doi:10.3389/fgene.2024.1400906)
Supplement: Supplementary file 1 [file Table1.DOCX]

## Supplementary Material

## Case Report: Charcot-Marie-Tooth Disease Caused by a De Novo *MORC2* Gene Mutation-Novel Insights into Pathogenicity and Treatment

1. **Supplementary Figures**


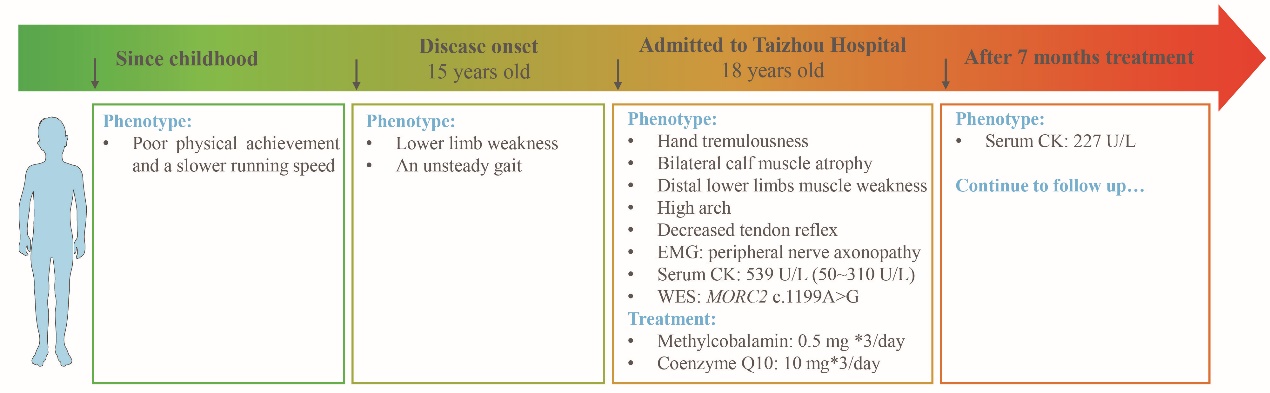


**Supplementary Figure 1**. The timeline of CMT disease progression.

**
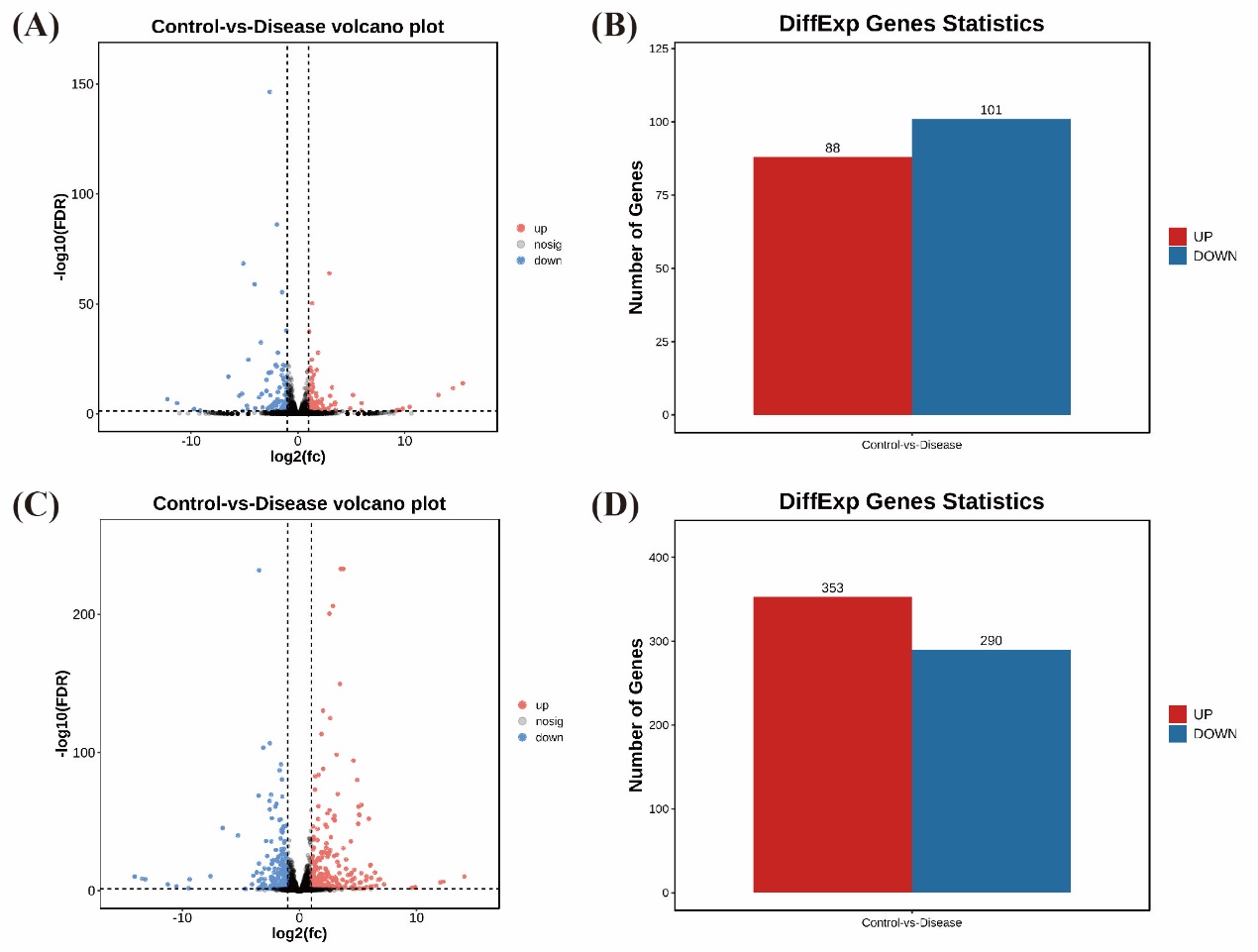
**

**Supplementary Figure 2**. Differentially transcribed genes (DEGs) in iPSCs and MNPs carrying the MORC2 p.Q400R. The transcriptomic data are acquired from the published literature *Zeng S, et al* (1). Employing the DESeq2 method, as introduced in the published study (*Zeng S, et al*) and facilitated by OmicShare Tools(2), we identified 189 DEGs in iPSCs (A, B) and 643 DEGs in MNPs (C, D) when comparing healthy controls and p.Q400R. Significantly differentially expressed genes were defined as those having an adjusted P-value < 0.05 and |log2(fold change)| ≥ 1.

**
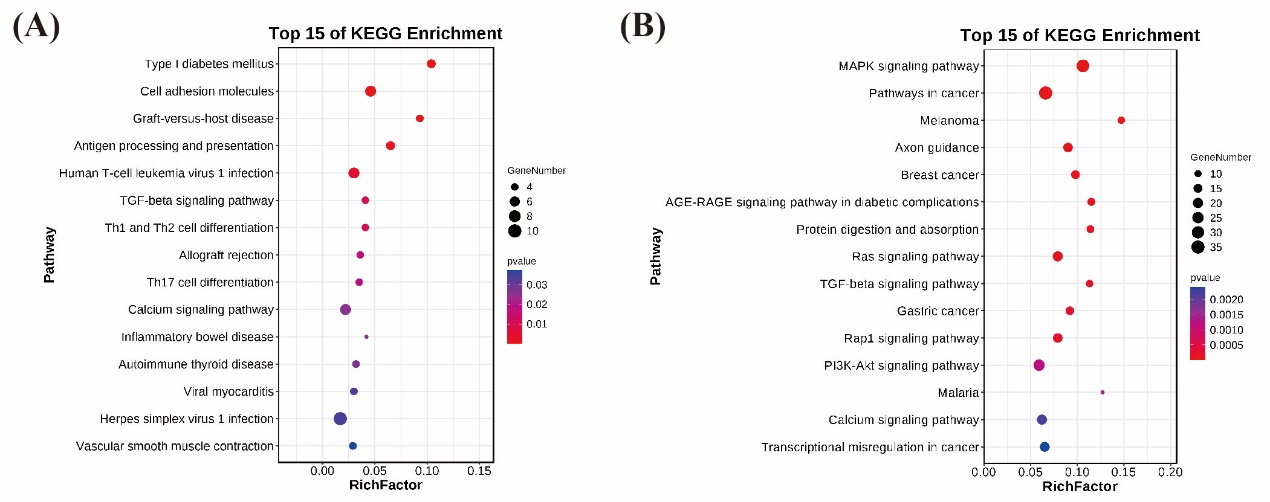
**

**Supplementary Figure 3**. Kyoto Encyclopedia of Genes and Genomes (KEGG) analysis of DEGs in iPSCs (A) and MNPs (B). (A, B) KEGG pathway terms enriched in DEGs between healthy controls and *MORC* p.Q400R.

1. **Supplementary reference**

1. Zeng S, Yang H, Wang B, Xie Y, Xu K, Liu L, et al. The MORC2 p.S87L mutation reduces proliferation of pluripotent stem cells derived from a patient with the spinal muscular atrophy-like phenotype by inhibiting proliferation-related signaling pathways. Neural Regen Res. 2024;19(1):205-11.

2. Mu H, Chen J, Huang W, Huang G, Deng M, Hong S, et al. OmicShare tools: A zero-code interactive online platform for biological data analysis and visualization. iMeta.n/a(n/a):e228.
